# Cardiovascular Assessment Tool for Breast Cancer Survivors and Oncology Providers: Usability Study

**DOI:** 10.2196/18396

**Published:** 2021-01-21

**Authors:** Kathryn E Weaver, Heidi D Klepin, Brian J Wells, Emily V Dressler, Karen M Winkfield, Zanetta S Lamar, Tiffany P Avery, Nicholas M Pajewski, W Gregory Hundley, Aimee Johnson, Eleanor C Davidson, Marcelo Lopetegui, Randi E Foraker

**Affiliations:** 1 Department of Social Sciences and Health Policy Wake Forest School of Medicine Winston-Salem, NC United States; 2 Department of Implementation Science Wake Forest School of Medicine Winston-Salem, NC United States; 3 Section on Hematology-Oncology Department of Internal Medicine Wake Forest School of Medicine Winston-Salem, NC United States; 4 Department of Biostatistics and Data Science Wake Forest School of Medicine Winston-Salem, NC United States; 5 Department of Family Medicine Wake Forest School of Medicine Winston-Salem, NC United States; 6 Department of Radiation Oncology Wake Forest School of Medicine Winston-Salem, NC United States; 7 Section on Cardiology Department of Internal Medicine Wake Forest School of Medicine Winston-Salem, NC United States; 8 Instituto de Ciencias e Innovación en Medicina Facultad de Medicina Clínica Alemana Universidad del Desarrollo Santiago Chile; 9 Institute for Informatics Washington University in St Louis School of Medicine St Louis, MO United States

**Keywords:** electronic health records, clinical decision support, usability testing, cardiovascular diseases, cancer survivors, breast cancer

## Abstract

**Background:**

Cardiovascular health is of increasing concern to breast cancer survivors and their health care providers, as many survivors are more likely to die from cardiovascular disease than cancer. Implementing clinical decision support tools to address cardiovascular risk factor awareness in the oncology setting may enhance survivors’ attainment or maintenance of cardiovascular health.

**Objective:**

We sought to evaluate survivors’ awareness of cardiovascular risk factors and examine the usability of a novel electronic health record enabled cardiovascular health tool from the perspective of both breast cancer survivors and oncology providers.

**Methods:**

Breast cancer survivors (n=49) recruited from a survivorship clinic interacted with the cardiovascular health tool and completed pre and posttool assessments about cardiovascular health knowledge and perceptions of the tool. Oncologists, physician assistants, and nurse practitioners (n=20) who provide care to survivors also viewed the cardiovascular health tool and completed assessments of perceived usability and acceptability.

**Results:**

Enrolled breast cancer survivors (84% White race, 4% Hispanic ethnicity) had been diagnosed 10.8 years ago (SD 6.0) with American Joint Committee on Cancer stage 0, I, or II (45/49, 92%). Prior to viewing the tool, 65% of survivors (32/49) reported not knowing their level for one or more cardiovascular health factors (range 0-4). On average, only 45% (range 0%-86%) of survivors’ known cardiovascular health factors were at an ideal level. More than 50% of survivors had ideal smoking status (45/48, 94%) or blood glucose level (29/45, 64%); meanwhile, less than 50% had ideal blood pressure (12/49, 24%), body mass index (12/49, 24%), cholesterol level (17/35, 49%), diet (7/49, 14%), and physical activity (10/49. 20%). More than 90% of survivors thought the tool was easy to understand (46/47, 98%), improved their understanding (43/47, 91%), and was helpful (45/47, 96%); overall, 94% (44/47 survivors) liked the tool. A majority of survivors (44/47, 94%) thought oncologists should discuss cardiovascular health during survivorship care. Most (12/20, 60%) oncology providers (female: 12/20, 60%; physicians: 14/20, 70%) had been practicing for more than 5 years. Most providers agreed the tool provided useful information (18/20, 90%), would help their effectiveness (18/20, 90%), was easy to use (20/20, 100%), and presented information in a useful format (19/20, 95%); and 85% of providers (17/20) reported they would use the tool most or all of the time when providing survivorship care.

**Conclusions:**

These usability data demonstrate acceptability of a cardiovascular health clinical decision support tool in oncology practices. Oncology providers and breast cancer survivors would likely value the integration of such apps in survivorship care. By increasing awareness and communication regarding cardiovascular health, electronic health record–enabled tools may improve survivorship care delivery for breast cancer and ultimately patient outcomes.

## Introduction

Cardiovascular health is of increasing concern to breast cancer survivors and their health care providers [1,2], since older, postmenopausal survivors are more likely to die of cardiovascular disease rather than of cancer [3,4]. Breast cancer survivors are at greater risk of death due to cardiovascular disease, compared to age-matched women without a history of breast cancer [5]. Chemotherapy (eg, anthracyclines), monoclonal antibody treatment, hormonal treatments, and radiation all heighten cardiovascular disease risk among survivors [1,6], further increasing cardiovascular disease susceptibility among cancer survivors [5,7,8]. Addressing cardiovascular health is critical for all breast cancer survivors, especially those who receive cardiotoxic treatments [2,9,10].

According to 1582 long-term cancer survivors surveyed in California, 62% were overweight or obese, 55% were hypertensive, 21% were diabetic, 18% were physically inactive, and 5% were current smokers [11]. An analysis of these California cancer registry data highlighted the possible role of shared risk factors in the development of both cancer and cardiovascular disease, reporting that cancer survivors tend to have multiple cardiovascular disease risk factors and that survivorship care often does not address these risk factors [11,12]. Early recognition and treatment of cardiovascular risk factors may be important during survivorship, as this increased risk of cardiovascular death is evident approximately 7 years postdiagnosis [2,5].

Despite Institute of Medicine recommendations for adequate prevention efforts and care coordination for cancer survivors [13-15], cardiovascular risk continues to be undertreated in this population [16,17]. The majority of National Cancer Institute Community Oncology Research Program oncologists we interviewed (11 of 14) in a pilot study [18] reported cardiovascular health discussions to be “somewhat” or “very” important. Yet in general survivorship settings, few referrals for cardiovascular care are made by oncologists to primary care providers and cardiologists for guideline-driven follow-up care [11,16,19,20].

The American Heart Association’s (AHA) definition of cardiovascular health comprises modifiable risk factors, which are scored according to Table 1. Improvements in cardiovascular health can reduce cardiovascular disease and breast cancer recurrence risk [21-26], and increasing patient and provider awareness can enhance cardiovascular health [13]. Most cancer survivors do not meet AHA’s healthy standards in multiple cardiovascular health components such as body mass index (BMI), physical activity, diet, smoking, blood pressure, cholesterol level, and glucose level [2,11,21].

**Table 1:** American Heart Association simple 7 measures of cardiovascular health, adapted from [27].

Measures			Poor health	Intermediate health	Ideal health
**Health behaviors **					
	Smoking status		Current	Former ≤12 months	Never or quit >12 months
	BMI		≥30 kg/m^2^	25-29.9 kg/m^2 ^	<25 kg/m^2^
	Physical activity		None	1-149 minutes/week moderate or 1-74 minutes/week vigorous or 1-149 minutes/week moderate and vigorous	≥150 minutes/week moderate or ≥75 minutes/week vigorous or ≥150 minutes/week moderate and vigorous
	Healthy diet score		0-1 components	2-3 components	4-5 components
**Health factors **					
	Total cholesterol level		≥240 mg/dL	200-239 mg/dL or treated to goal	<200 mg/dL
	Blood pressure		Systolic ≥140 mm Hg or Diastolic ≥90 mm Hg	Systolic 120-139 mm Hg or Diastolic 80-89 mm Hg or treated to goal	Systolic <120 mm Hg Diastolic <80 mm Hg
	**Blood glucose level**				
		Fasting plasma glucose	≥126 mg/dL	100-125 mg/dL or treated to goal	<100 mg/dL
		Hemoglobin A_1c_	≥6.5%	5.7%-6.4% or treated to goal	≤5.6%

**Figure 1:**
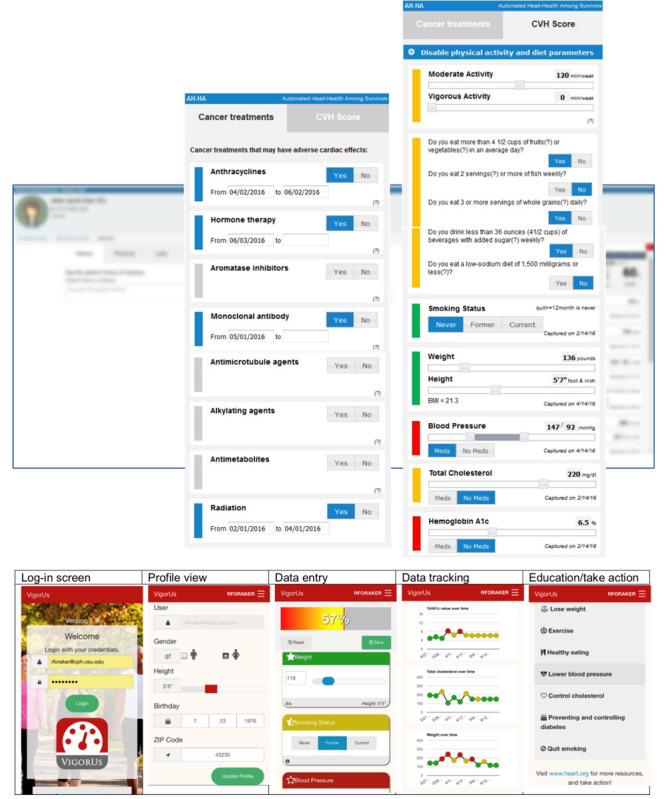
Adapted AH-HA tool (top) and the Vigor-Us tool (bottom).

Clinical decision support can provide relevant data to the point-of-care to prompt appropriate disease management and referrals [28]. Our team has previously developed, implemented, and evaluated a cardiovascular health assessment tool, Stroke Prevention in Health care Delivery Environments (SPHERE), in the primary care setting [29,30]. Use of SPHERE resulted in improved BMI and diabetes status in the interventional primary care clinic but not the control clinic [31]. We refined this tool based upon feedback received from qualitative interviews with oncologists [18] and added information about receipt of potentially cardiotoxic cancer treatments. For this study, we evaluated the acceptability of the new Automated Heart-Health Assessment tool (AH-HA, Figure 1) among oncology providers and the Vigor-Us mobile app (Figure 1) among breast cancer survivors. We hypothesized that the majority of survivors and oncology providers would express positive views about the tools and their use in the cancer survivorship setting.

The AH-HA tool was embedded within a simulated electronic health record environment and was intended to be used mainly with a cursor pointer (mouse). The Vigor-Us tool was a responsive web app suitable for both touch and click interactions, with larger interactivity components. AH-HA did not collect any data from the interface, whereas Vigor-Us collected all the information entered during the authenticated sessions (authenticated users, secure sockets layer–encrypted database).

## Methods

### Ethical Approval and Informed Consent

This study was approved by the Wake Forest Health Sciences Institutional Review Board. All procedures performed in studies involving human participants were in accordance with the ethical standards of the institutional research committee and with the 1964 Helsinki declaration and its later amendments or comparable ethical standards. The Wake Forest Health Sciences Institutional Review Board approved the study with a waiver of written informed consent.

### Study Eligibility and Data Collection

Eligible survivors included those who were at least 21 years of age, diagnosed with nonmetastatic breast cancer, and at least 3 months after potentially curative cancer treatment (ie, surgery, chemotherapy, or radiation), excluding maintenance hormonal therapy. Additional inclusion criteria included no current evidence of disease or a history of cancer recurrence, a working email address, and ability to read medical information in English. Survivors were ineligible for the study if they had visual impairments that prohibited them from viewing material on a tablet device or if they were enrolled in hospice care or had a life expectancy less than 6 months. Survivors were identified through clinic appointment schedules and contacted by a research member prior to their appointment by telephone or immediately before their appointment in the waiting room.

Eligible providers included medical, radiation, gynecologic, and surgical oncologists; nurse practitioners, and physician assistants who provided survivorship care to posttreatment cancer survivors. A list of eligible oncologists, nurse practitioners, and physician assistants was procured from the oncology service line administrators, and providers were emailed an invitation to participate.

All participants provided informed consent prior to participation, and the study was approved by the Wake Forest Health Sciences Institutional Review Board (number 37786). Survivor participants completed a baseline survey, viewed the Vigor-Us tool (Figure 1) with their cardiovascular health information on a tablet computer with the study research coordinator, and then completed a brief postsurvey. The total research visit time was 15 to 20 minutes. Separate from the survivor assessment, provider participants were provided with a prototype of the enhanced AH-HA tool (Figure 1) on a tablet computer, introduced to the manipulation of slider bars and buttons, and asked to use the tool as they might with a cancer survivor. Providers also completed brief assessments before and after viewing the tool. Both survivor and provider participants received a US $10 gift card.

### Cardiovascular Health Assessment Tools

The AH-HA tool (Figure 1) visualizes data regarding the AHA Simple 7 modifiable cardiovascular heath factors—BMI, smoking status, blood pressure, total cholesterol level, hemoglobin A_1c_ or fasting glucose level, healthy diet, and physical activity [27]—to promote discussions at the point of care between breast cancer survivors and oncology providers. AH-HA was adapted from the SPHERE [30] primary care tool to include information about receipt of potentially cardiotoxic chemotherapies (ie, anthracyclines, antimetabolites, hormone therapy, aromatase inhibitors, monoclonal antibodies, antimicrotubule agents, alkylating agents, and radiation) [32] and was designed to be integrated with electronic health records (EHR) using Fast Healthcare Interoperability Resources [33]. Breast cancer survivors viewed a patient-facing version of the SPHERE tool designed for personal computers and mobile devices, the Vigor-Us app (Figure 1). This app did not contain information about receipt of potentially cardiotoxic chemotherapies because our clinical advisory group felt that this information was best discussed with a medical provider.

### Measures

Survivor cardiovascular risk information abstracted from the medical record include weight, height, smoking status, blood pressure, total cholesterol level, and hemoglobin A_1c_ level; self-reported factors included smoking, physical activity, and diet (Table 1). Survivor knowledge of cardiovascular health and perceived importance and appropriateness of heart health discussions during oncology care were evaluated with 6 questions assessing confidence in understanding risk of heart disease, understanding steps needed to improve heart health, perception that cancer (or heart disease) poses a risk to health, and desire to talk to a provider (oncologist or primary care provider) about heart health. Survivors were also asked about the numerical value of each heart health factor (with “I don’t know” as an option) and to rate each health factor as high (poor health), somewhat high (intermediate health), or low-risk (ideal health) according to Table 1. Following their use of the tool, survivors completed the same 6 preassessment questions along with 3 additional questions reflecting acceptability of heart health discussions with oncologists prior to, during, and after treatment completion. Survivor tool acceptability was assessed with 5 questions on a 5-point Likert scale (strongly agree to strongly disagree) regarding liking the tool, helpfulness, ease of understanding, picture/diagram improved understanding, and desire to use this tool with oncologist. Survivors also reported gender, race and ethnicity, years of education, internet and email usage, and health literacy [34].

Provider self-reported demographic and practice data included gender, race/ethnicity, years in practice, and percentage of time spent in patient care. Provider usability was assessed using 6 questions utilized in our previous study of general internal medicine physicians [29] assessing useful information, promotion of effectiveness, ease in accessing needed information, information meets needs, easy to use, and useful format. These questions were rated on a 7-point Likert scale from strongly agree to strongly disagree. Three questions reflecting potential use of tool prior to, during, and after treatment completion were rated on a 4-point Likert scale (never, almost, always, almost always).

### Statistical Analyses

For this pilot study, the sample size for the oncology provider survey (n=20) is driven primarily by feasibility concerns. For the survivor survey, we estimated power to test the hypothesis that responses to each Likert scale question are generally positive, which we defined as testing the alternative hypothesis that the mean score for each question is greater than 3.5 (where a score of 3 denotes a neutral response to the question). Assuming a sample of 50 survivors and a standard deviation of 1.0, we will have >80% power provided the true mean score for a particular question is 3.9 or greater (roughly corresponds to an average response of agree).

We conducted descriptive analyses and summarized oncology provider and survivor demographics and survey responses with counts and percentages. Providers’ responses were assessed on a 7-point Likert scale from strongly disagree to strongly agree; we categorized responses of 5-7 as agreeing. Survivors’ responses were assessed on a 5-point Likert scale from strongly agree to strongly disagree; responses of agree or strongly agree were categorized as agreeing. Wilcoxon signed rank tests were used to compare breast cancer survivors’ knowledge regarding their cardiovascular risk factors and perceived importance of cancer and heart disease before and after viewing the tool. Comparisons were made individually for each of the 6 questions included on the questionnaire about cardiovascular risk factor knowledge and perceived importance and appropriateness of heart health discussions during oncology care. Sidak correction for multiple testing were utilized due to the 6 questions; *P* values <.0085 were considered significant for these outcomes. We calculated the percent of survivors who reported “I don’t know” for each cardiovascular risk factor. Among those who did respond with a value for their risk factor, we calculated percent agreement between categorization of objective EHR data and the survivor’s subjective assessment. Finally, we present survivor and provider data on usability of the tools. Specifically, we calculated the percent of survivors and providers who agreed or strongly agreed with the usability questions, and we presented data on the preferred timing of the intervention according to survivors and providers.

## Results

### Sociodemographic and Health Characteristics of Breast Cancer Survivors

We enrolled 49 breast cancer survivors (Table 2). An additional 13 survivors were screened and not enrolled (4 did not have an email address, 6 were not interested, and 3 could not stay after an appointment). The majority of enrolled survivors (92%) had an early-stage cancer and were on 11 years postdiagnosis (mean 10.8 years, SD 6); all received surgical treatment, 55% (27/49) received chemotherapy, and 69% (34/49) received radiation. With regards to receipt of potentially cardiotoxic cancer treatments, one-third (17/49, 35%) had received treatment with an anthracycline; almost half received hormone therapy (24/49, 49%); 45% (22/49) received aromatase inhibitors, 6% (3/49) received monoclonal antibodies, 29% (14/49) received antimicrotubule agents, 43% (21/49) received alkylating agents, and 8% (4/49) received antimetabolites. Almost half of survivors (23/49, 47%) reported graduating from college, and 96% (47/49) reported adequate health literacy. Most had a cell phone (47/49, 96%), used the internet (43/49, 88%), and used email almost every day (34/49, 69%). Almost all, survivors (47/49, 96%) completed the postvisit assessment and provided cardiovascular health tool usability data.

**Table 2:** Characteristics of breast cancer survivor (n=49) and oncology provider (n=20) participants for usability testing of the AH-HA tool.

Characteristic		Breast cancer survivors (n=49)	Oncology providers (n=20)
**Age (years), n (%)**			
	<65	28 (57)	N/A^a^
	≥65	21 (43)	N/A
**Sex, n (%)**			
	Female	49 (100)	12 (60)
	Male	0 (0)	8 (40)
**Race, n (%)**			
	White	41 (84)	14 (70)
	Black or African American	4 (8)	2 (10)
	Asian	1 (2.0)	1 (5)
	Southeast Asian	0 (0)	3 (15)
	American Indian	0 (0)	1 (5)
	More than one race	2 (4)	0 (0)
	Unknown	1 (2)	0 (0)
Hispanic ethnicity, n (%)		2 (4)	2 (10)
Time since cancer diagnosis (years), mean (SD)		10.8 (6.0)	N/A
**Cancer treatment received, n (%)**			
	Surgery	49 (100)	N/A
	Chemotherapy	27 (55)	N/A
	Radiation	34 (69)	N/A
**AJCC^b^ stage, n (%)**			
	0	4 (8)	N/A
	I	23 (47)	N/A
	II	18 (37)	N/A
	III	4 (8)	N/A
**Education level, n (%)**			
	≤High school	7 (14)	N/A
	Some college	19 (40)	N/A
	College graduate	23 (47)	N/A
**Email use every day or almost every day, n (%) **			
	Yes	34 (69)	N/A
	No	15 (31)	N/A
**Internet use past 30 days, n (%)**			
	Yes	43 (88)	N/A
	No	6 (12)	N/A
**Adequate health literacy, n (%)**			
	Yes	47 (96)	N/A
	No	2 (4)	N/A
**Provider type, n (%)**			
	Physician (MD or DO)	N/A	14 (70)
	Physician assistant/nurse practitioner	N/A	6 (30)
**Years in practice, n (%)**			
	≤5	N/A	8 (40)
	6-10	N/A	4 (20)
	≥11	N/A	8 (40)
**Oncology specialty, n (%)**			
	Hematology	N/A	13 (65)
	Radiation	N/A	1 (5)
	Surgical	N/A	6 (30)
**Time spent in direct patient care, n (%)**			
	≤50%	N/A	4 (20)
	51-75%	N/A	7 (35)
	>75%	N/A	9 (45)

^a^N/A: not applicable.

^b^AJCC: American Joint Committee on Cancer.

### Cardiovascular Health and Awareness of Breast Cancer Survivors

**Figure 2:**
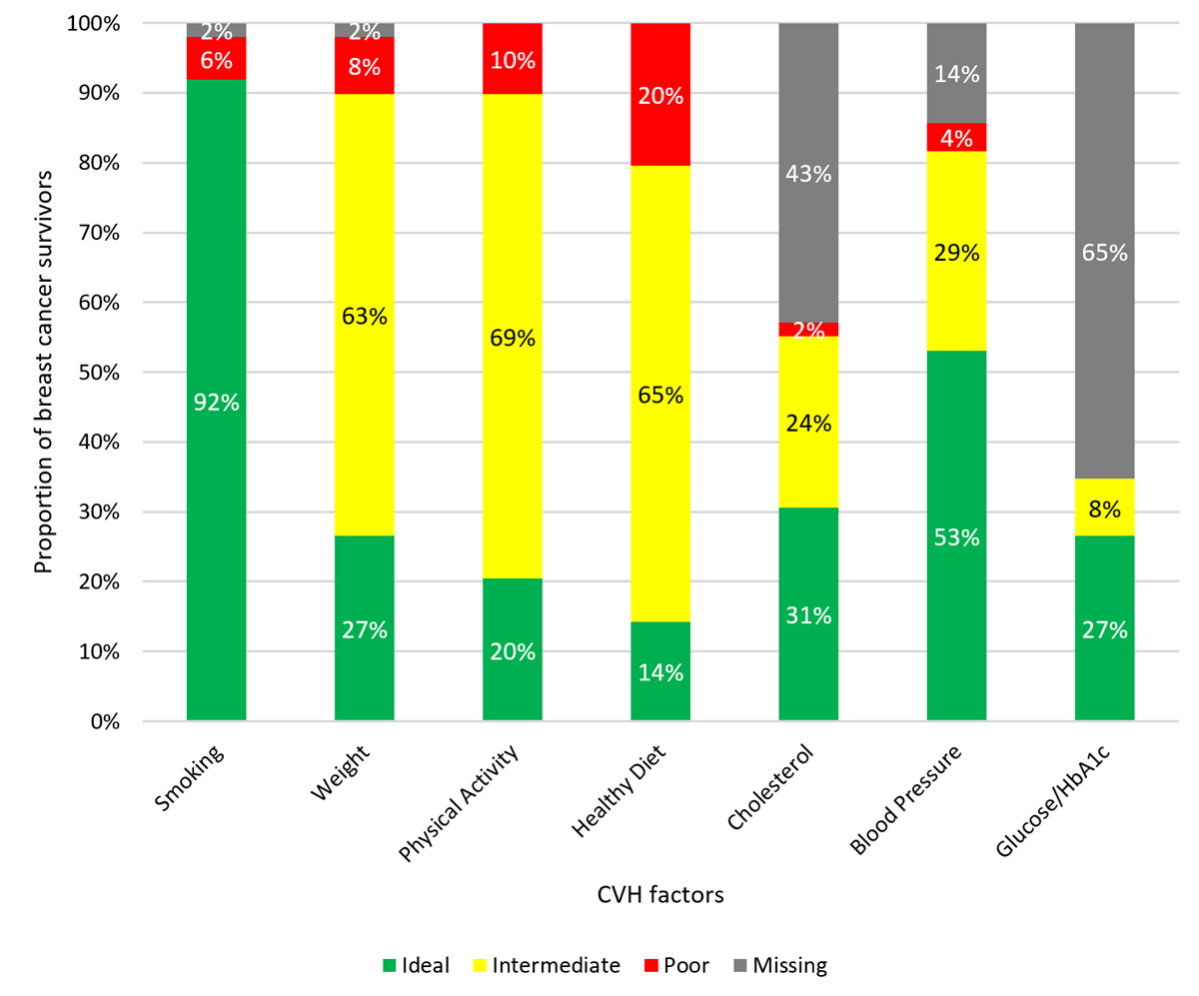
Proportion of breast cancer survivors (n=49) reporting poor (red), intermediate (yellow), ideal (green), and missing (gray) cardiovascular health factors. CVH: cardiovascular health.

Prior to viewing the tool, 90% of survivors (44/49) agreed that cancer posed a risk to their health, and 84% (41/49) agreed that cardiovascular disease posed a risk to their health. On average, only 45% (range 0%-86%) of survivors’ known cardiovascular health factors were reported to be at an ideal level. More than 50% of survivors reported smoking status (45/49, 92%) and blood pressure (26/49, 53%) in the ideal category; less than one-third reported BMI, diet, and physical activity in the ideal range (Figure 2).

**Figure 3:**
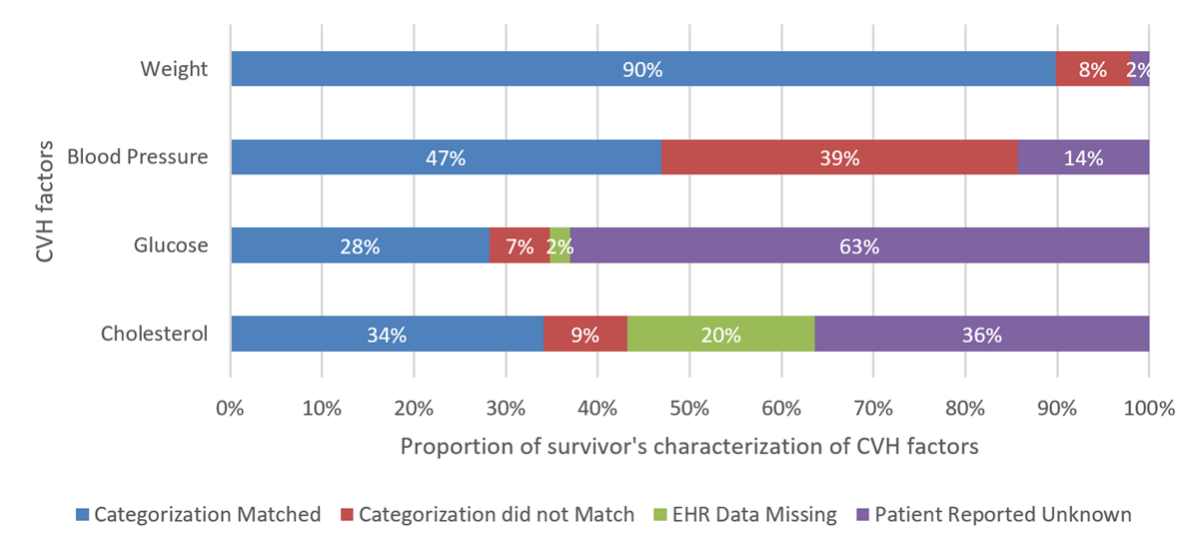
Survivor characterization of cardiovascular health factors. CVH: cardiovascular health; EHR: electronic health record.

Prior to viewing the tool, 24% of survivors (12/49) expressed strong agreement that they understood their risk of cardiovascular disease; 58% (28/49) agreed. Yet 65% (32/49) reported not knowing the level for one or more cardiovascular health factors (range 0-4). Cardiovascular risk factors most likely to be self-reported as “not known” (Figure 2) included hemoglobin A_1c_ (44/49, 90%), blood glucose level (32/49, 65%), cholesterol level (21/49, 43%), blood pressure (7/49, 14%), and BMI (1/49, 2%). When comparing concordance between the EHR and self-report for categorization of cardiovascular health factors as ideal vs nonideal among survivors who knew the categorization of their factor, 90% of survivors (44/49) were concordant for BMI, 47% (23/49) were concordant for blood pressure, 28% (14/49) were concordant for blood glucose level, and 34% (17/49) were concordant for cholesterol level (Figure 3).

### Usability of the Tool Among Breast Cancer Survivors

**Figure 4:**
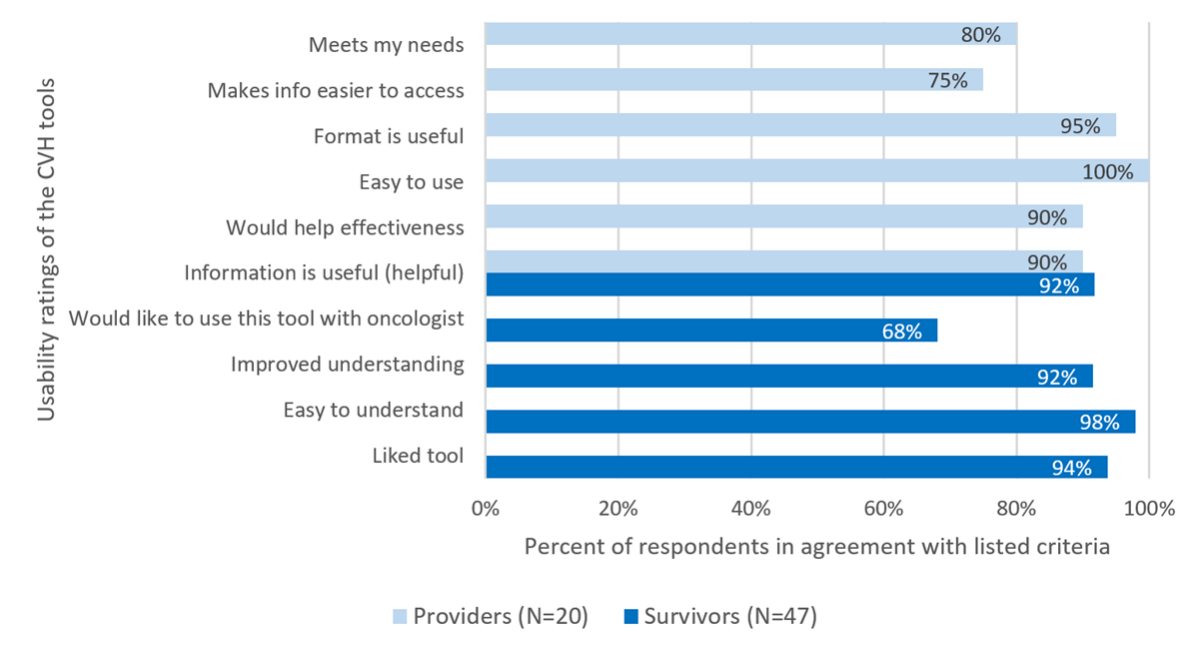
Usability ratings of the tools among breast cancer survivors and oncology providers. CVH: cardiovascular health.

Usability ratings of the tool by breast cancer survivors are shown in Figure 4. The majority of breast cancer survivors thought the tool was easy to understand (48/49, 98%), improved their understanding (45/49, 92%), and was helpful (45/49, 92%); 94% (46/49) liked the tool and agreed oncologists should discuss heart health during survivorship care. A majority (34/49, 69%) would like to use the tool with their oncologist at a future appointment. There were no differences in usability statistics by those 65 years and older versus those younger than 65 years.

**Figure 5:**
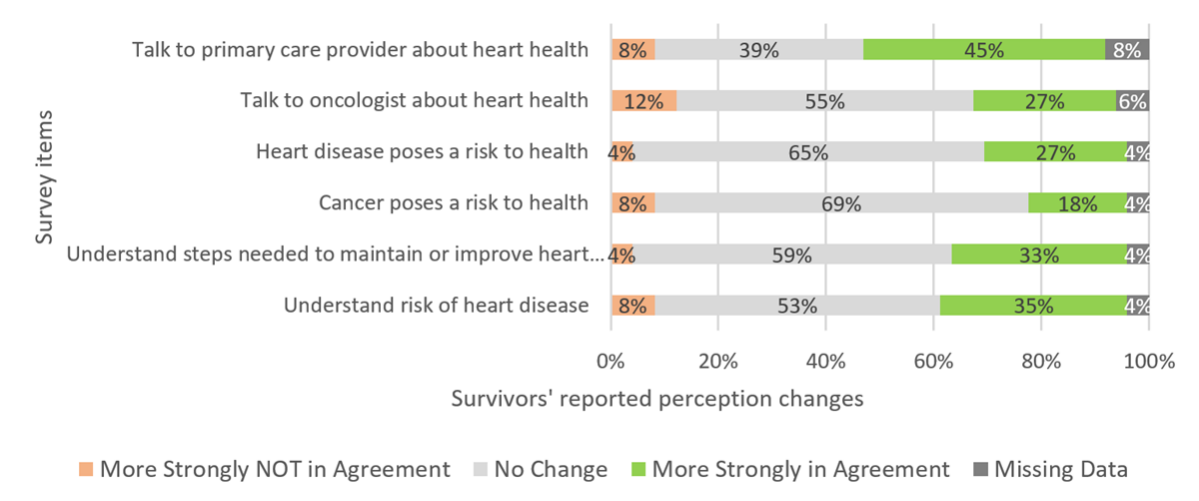
Proportion of breast cancer survivors (n=47) whose cardiovascular health perceptions changed before and after viewing the assessment tool.

We also assessed survivors’ perception of cardiovascular risk before and after viewing the tool (Figure 5). For all variables, survivors reported that they were in stronger agreement with the statements after viewing the tool (Figure 5). Significant changes were observed for understanding of cardiovascular risk (*S*=–65, *P*=.009), understanding steps to improve cardiovascular health (*S*=–70.5, *P*<.001, perception of health risk from cardiovascular disease (*S*=–45, *P*=.007), and desire to discuss cardiovascular risk with a primary care provider (*S*=–121, *P*<.001). There was no significant change in perception of health risk from cancer or desire to discuss cardiovascular risk with an oncologist.

### Sociodemographic and Practice Characteristics of Providers

We enrolled 14 physicians, 2 physician assistants, and 4 nurse practitioners; 60% (12/20) were female, 70% (14/20) were White, 10% (2/20) were Black, and 10% (2/20) identified as Hispanic or Latino (Table 2). Hematology oncology was defined as the practice specialty for 65% of providers (13/20), 60% (12/20) had been practicing as an attending for more than 5 years, and 80% (16/20) spent more than 50% of their time in direct patient care. Only 50% (10/20) reported usually or always talking to their posttreatment patients about cardiovascular health, and 35% (7/20) usually or always initiated discussion about cardiovascular health with posttreatment patients. However, 95% (19/20) reported it was somewhat or very important to discuss cardiovascular health with posttreatment patients. About half of providers (9/20, 45%) reported a high level of comfort with cardiovascular health discussions.

### Usability of the Tool Among Oncology Providers

Usability ratings of the tool by providers are shown in Figure 4. Most providers agreed the tool provided useful information (18/20, 90%), would help their effectiveness (18/20, 90%), was easy to use (20/20, 100%), and presented information in a useful format (19/20, 95%); and 85% of providers (17/20) reported they would use the tool most or all of the time when providing survivorship care, with 50% (10/20) reporting the same for initial treatment planning and 45% (9/20) during active treatment.

## Discussion

### Principal Results

Overall, our results suggest both the need for and suitability of a tailored cardiovascular health assessment tool to heighten awareness of cardiovascular health among oncology providers and breast cancer survivors. We present the first usability data from breast cancer survivors and oncology providers on the usability of EHR-integrated cardiovascular health assessment tools. On average, only 45% of breast cancer survivors’ known cardiovascular health factors were at an ideal level, most survivors did not know the value or categorization of at least one of their cardiovascular health factors, and 94% of survivors (46/49) thought oncologists should discuss heart health during survivorship care. Nearly all providers indicated that it was either somewhat important or very important to discuss cardiovascular health with posttreatment patients. However, less than half of providers reported a high level of comfort with cardiovascular health discussions, and only half reported usually or always talking to their posttreatment patients about cardiovascular health. Usability data from providers and survivors demonstrate positive perceptions of the cardiovascular health apps; 85% of providers (17/20) reported they would use the tool most or all of the time when providing survivorship care. Thus, we conclude that clinical decision support tools such as AH-HA have potential to provide relevant data to providers at the point of care to initiate discussions and prompt appropriate referrals to primary care and cardiology—settings in which cardiovascular health can be managed effectively.

The use of the AH-HA and Vigor-Us tools are one strategy for improving risk assessment and personalized cardiovascular disease prevention in cancer survivorship programs, a research priority identified by the AHA [2]. A majority of breast cancer survivors did not know one or more of their cardiovascular health risk factors, despite a majority expressing agreement before viewing the tool that they understood their risk of heart disease. In particular, knowledge gaps exist among survivors with respect to their hemoglobin A_1c_ and cholesterol values, which are strong independent predictors of cardiovascular disease [35]. Self-reported understanding of cardiovascular risk increased among survivors with use of the tool, and survivors increased their interest in discussing their heart health with primary care providers following the use of the tool. This increased awareness and interest may facilitate linking survivors back into primary care so these risks can be addressed.

### Comparison With Prior Work

Our results are consistent with our previous evaluation of general cardiovascular health clinical decision support in the primary care setting. In our previous study [29], providers indicated that the content and the accuracy of the tool met their needs always or most of the time. Primary care providers felt the tool was clear and presented data in a useful format, was easy to use and user-friendly, and provided up-to-date information in a timely manner [29].

### Limitations

Limitations of this study include the smaller sample size, nonrandomized usability assessment, single-institution setting, and the absence of data regarding the impact of the tool on cardiovascular health and health care utilization. Although we focused on breast cancer survivors in this usability study, the tool may also be appropriate for other survivor populations who have significant competing risk from cardiovascular disease. Future testing of this tool should take place in more diverse multi-institutional settings.

### Conclusions

The AH-HA point-of-care EHR-based visualization tool brings together personalized cardiovascular health and contextual cancer treatment data to address potential gaps in breast cancer survivorship care. Our previous SPHERE study [29] suggested that cardiovascular health clinical decision support tools are well-received in the primary care setting. Findings from the current study suggest that oncology providers and breast cancer survivors would benefit from and value the integration of cardiovascular health clinical decision support apps in survivorship care. A newly initiated study will test the effectiveness and implementation of the AH-HA app in a clinic-randomized trial in community oncology practices.

## Abbreviations

AHA: American Heart Association
AH-HA: Automated Heart-Health Assessment
EHR: electronic health record
SPHERE: Stroke Prevention in Healthcare Delivery Environments
